# LymPHOS 2.0: an update of a phosphosite database of primary human T cells

**DOI:** 10.1093/database/bav115

**Published:** 2015-12-23

**Authors:** Tien Dung Nguyen, Oriol Vidal-Cortes, Oscar Gallardo, Joaquin Abian, Montserrat Carrascal

**Affiliations:** ^1^CSIC/UAB Proteomics Laboratory, Instituto De Investigaciones Biomédicas De Barcelona-Consejo Superior De Investigaciones Científicas (IIBB-CSIC/IDIBAPS), Rosellón 161 6a Planta, Barcelona E-08036, Spain

## Abstract

LymPHOS is a web-oriented database containing peptide and protein sequences and spectrometric information on the phosphoproteome of primary human T-Lymphocytes. Current release 2.0 contains 15 566 phosphorylation sites from 8273 unique phosphopeptides and 4937 proteins, which correspond to a 45-fold increase over the original database description. It now includes quantitative data on phosphorylation changes after time-dependent treatment with activators of the TCR-mediated signal transduction pathway. Sequence data quality has also been improved with the use of multiple search engines for database searching. LymPHOS can be publicly accessed at http://www.lymphos.org.

**Database URL:** http://www.lymphos.org.

## Introduction

Regulation of cell function is mediated by changes in protein activity, protein stability and protein–protein interactions, through the action of a wide range of post-translational modifications (PTMs). Over 460 PTMs are described in the Uniprot database (http://www.uniprot.org/docs/ptmlist), ranging from the attachment of small compounds such as acetyl groups (acetylation) or phosphate groups (phosphorylation), to the addition of larger molecules or peptide chains as in the cases of ubiquitination and glycosylation. The technological advances in recent years, especially in mass spectrometry, have allowed a more effective study of the proteome. In 2008, UniProtKB/Swiss-Prot generated the first draft of the human proteome including 20 000 protein-coding genes. In 2013, spectrometric data repositories such as PRIDE accumulated more than 30 000 experiments with nearly 7 million unique peptides identified in different species (1). The establishment of these databases has promoted many initiatives such as the Human Proteome Project (HPP), which has among its objectives to sequence all proteins encoded in the human genome (including modified forms), as well as to characterize protein interaction networks and develop new specific antibodies (2).

While the sequencing of the human proteome is at a well advanced stage, the case for PTM mapping remains challenging. The technical issues of PTM analysis make their coverage level still very low (3). The characterization of these modifications is, however, vital for understanding the cellular mechanisms involved in disease. The important role of these processes in practice is evidenced by the high number of regulatory modified proteins related to diseases that are therapeutic targets of current or developing drugs (4).

One of the most studied PTMs is protein phosphorylation. Characterizing phosphoproteome components and their phosphorylation profiles in different conditions is necessary to develop new drugs modulating the activity of kinases and phosphatases. The importance of this area is reflected by the presence of 150 kinase inhibitors currently in clinical trials, on top of the 20 that have already been approved (5). This area alone is estimated to involve a 30% of R&D expenditures in the pharmaceutical industry.

The LymPHOS database was created in 2008 containing 342 p-sites from human primary T-lymphocytes (6). To date, we have identified 15 566 phosphorylation sites in a total of 8273 unique phosphopeptides belonging to 4937 proteins. About half of these sites have not been annotated in UniProt experimentally or by similarity and over 200 are neither described in PhosphoSite (http://www.phosphosite.org), one of the most complete p-site collections available. Additionally, LymPHOS contains quantitative information about changes in the phosphoproteome after cell activation with Phorbol 12-myristate 13-acetate (PMA) and ionomycin or with anti-CD3/CD28 monoclonal antibodies. To our knowledge, there are no other resources dedicated to phosphoproteome characterization of T-cells. Management of LymPHOS is now achieved through an automated workflow that includes MS data filtering, sequence identification by different search engines, phosphopeptide quantification after time-dependent treatment, accurate p-site assignation, and mass spectra visualization. This report is a brief description of the improvements and current status of this unique database.

## Methods

### Sample preparation

A total of 20 different qualitative and 11 quantitative experiments are included in the database (see Experimental section in the Lymphos2 website). In all cases, the starting material were pools of T cells purified from 4 to 5 healthy donors. For qualitative experiments one pool was used, while quantitative experiments included two biological replicates so that two different pools (i.e. 8–10 donors) were utilized per experiment. Lymphocytes from each donor were isolated from buffy coats through a density gradient centrifugation using Ficoll-Paque (GE, Uppsala, Sweden), followed by three washing steps to remove unwanted cellular contaminants and a 60 min plastic-adherence culture to remove monocytes as described elsewhere (7). Typically, a purity of ca. 80% in CD3+ T lymphocytes is achieved with this method. Cell stimulations were carried out with PMA/Ionomycin or with anti-CD3/anti-CD28 antibodies as previously described (8, 9). Protein extracts were digested with trypsin following standard procedures or using the FASP method (10). For quantitative purposes, tryptic peptides were labeled with iTRAQ or TMT following manufacturer’s instructions. Samples were then desalted by solid phase extraction (500 mg C18 Sep-Pack cartridges, Waters, MA) and fractionated by strong cation chromatography (SCX) using a Polysulfoethyl A TM, 100 × 2.1 mm, 5 µm, 200 Å column. (PolyLC, Columbia, MD). Each SCX fraction was then desalted by solid phase extraction (15 mg, C18, Varian), evaporated to near dryness and brought up to 200 µL with 250 mM acetic acid/30% acetonitrile for subsequent phosphopeptide enrichment. Phosphorylated peptides were enriched using immobilized metal affinity chromatography (IMAC) and titanium dioxide columns (TiO_2_) as performed in our previous studies (7, 11).

### Mass spectrometric analysis

All the IMAC and TiO_2_ fractions were analyzed separately by LC-MS*^n^* using an LTQ linear ion trap or an LTQ-Orbitrap XL system equipped with a microESI ion source (ThermoFisher, San Jose, CA). For qualitative studies, a full MS scan followed by eight MS/MS scans on the most abundant precursor signals were acquired. For quantitative purposes, the three more abundant precursors from each full MS were submitted to four MS/MS analyses (three PQD and one CID scan) in the linear ion trap. Eight precursors per full scan were selected in the case of the LTQ-Orbitrap each submitted to two different MS/MS analyses (one CID and one HCD). In all cases, a subsequent MS^3^ scan was performed when a neutral loss of −49, −32.7 or −24.5 (loss of H_3_PO_4_ for the +2, +3 and +4 charged ions, respectively) was detected among the 10 most intense ions in the CID MS/MS spectra. MS^3^ scans allow identification of peptides with poor MS^2^ sequence data in qualitative analyses. In quantitative analyses, MS^3^ scans also allow to assign peptides with insufficient CID MS^2^ data but with valid TMT or iTRAQ reporter ion data from the corresponding PQD or HCD scans.

### Database search and phosphopeptide validation

The identification of phosphopeptides from mass spectra was carried out following an automatic workflow developed in the laboratory, which is based on the use of different search engines in parallel (11). Using this strategy, only peptide assignments pointed by at least two search engines are considered as positive identifications. In order to generate a well-matched generic input for the distinct search engines (Sequest, OMSSA, EasyProt, Phenyx or PEAKS) the original mass spectrometric data files (in the proprietary Thermo Scientific .raw binary format) were converted and split into two separate Mascot Generic Format (MGF) files (for MS^2^ and MS^3^ data, respectively) using EasierMgf (12). When processing quantitative data, EasierMgf also inserts the intensities of the ions in the 100–150 Da range (which contains the TMT or iTRAQ reporter ions) from the PQD or HCD spectra into the corresponding CID spectra. All searches were performed allowing a maximum of +4 charges for precursor ions and MS^2^ and MS^3^ spectra were searched independently. Peptide mass tolerance was set to 2 Da and 20 ppm for linear ion trap and LTQ-Orbitrap, respectively; fragment tolerance was set to 0.8 Da; enzyme was set to trypsin, allowing up to one missed cleavage; static modification was carbamidomethylated cysteine (+57 Da); dynamic modifications were methionine oxidation (+16 Da) and phosphorylation on Ser, Thr and Tyr (+80 Da). In MS^3^ searches, dehydration of Ser and Thr was also taken into account.

Data from each search engine was aligned, homogenized, and integrated using the Integrator software (12). Integrator also determines the most probable phosphorylation site location from the corresponding MS/MS data according to the Q-Ascore algorithm. P-sites with Q-Ascore higher than 19 were considered of high confidence. Integrator produces an output file (in JSON format) containing mass spectrometric and identification information and the adequate structure to be uploaded to the LymPHOS database.

### Quantitative analysis

TMT or iTRAQ reporter ion intensities were used for quantitative analysis of the peptides identified by either MS^2^ or MS^3^. The capability of using MS^3^ identifications with MS^2^ derived quantification data is probably a unique characteristic of our tools. Before the analysis, data was normalized using the median of non-phosphorylated peptide intensities. All of these steps are automatically performed by our in-house developed PQuantifier software. This application uses the normalized reporter ion intensity values in order to calculate the ratio between each activation time and its respective control. For each identified spectrum, PQuantifier assigns missing values, averages the intensities of duplicate reporter ions, and calculates the ratios for the different time points relative to the control. For each experiment, PQuantifier normalizes these ratios relative to the distribution of non-phosphorylated peptides and calculates the peptide average ratios along the activation experiments. PQuantifier finally applies a *t*-test to determine which peptides show significant changes. The final results of the PQuantifier analysis, in JSON format, are uploaded onto the LymPHOS database.

### Biological analysis

KEGG pathway analysis. Proteins for which one or more sites were detected phosphorylated in resting or activated cells were mapped to the KEGG pathways (13) using DAVID Bioinformatics Resources 6.7 (http://david.abcc.ncifcrf.gov) (14) and the whole *Homo sapiens* genome as background.

Gene Ontology analysis. A Panther Database (version 10.0 Released 2015-05-15) statistical overrepresentation test (release 20150430) was performed on the full LymPHOS2 phosphoprotein contents and on the phosphoprotein subset containing regulated p-sites [http://pantherdb.org/ (15, 16)]. The test was carried out using the GO-slim terms for cellular compartment, molecular function and biological process. The reference protein list was the full *Homo sapiens* proteome.

### Database and web application structure

The LymPHOS web application consists of a relational database and a web interface that allows data submission, querying and visualization. The database uses MySQL (http://www.mysql.com) as a relational database management system. The web interface has been developed in Python (http://www.python.org) using the Django web framework (https://www.djangoproject.com), as well as other well tested Python libraries, such as matplotlib (http://matplotlib.org) and numpy (http://www.numpy.org) for the mass spectrometry data visualization and analysis.

Additionally, several other small Python scripts were created to populate the LymPHOS database with metadata (experimental conditions, MIAPE data) and to export and summarize its contained information. All source code for LymPHOS, PQuantifier and the accessory scripts are available under an Open Source license, and can be freely downloaded from our source code repository hosted at Bitbucket (https://bitbucket.org/lp-csic-uab).

## Results and discussion

**Figure 1:**
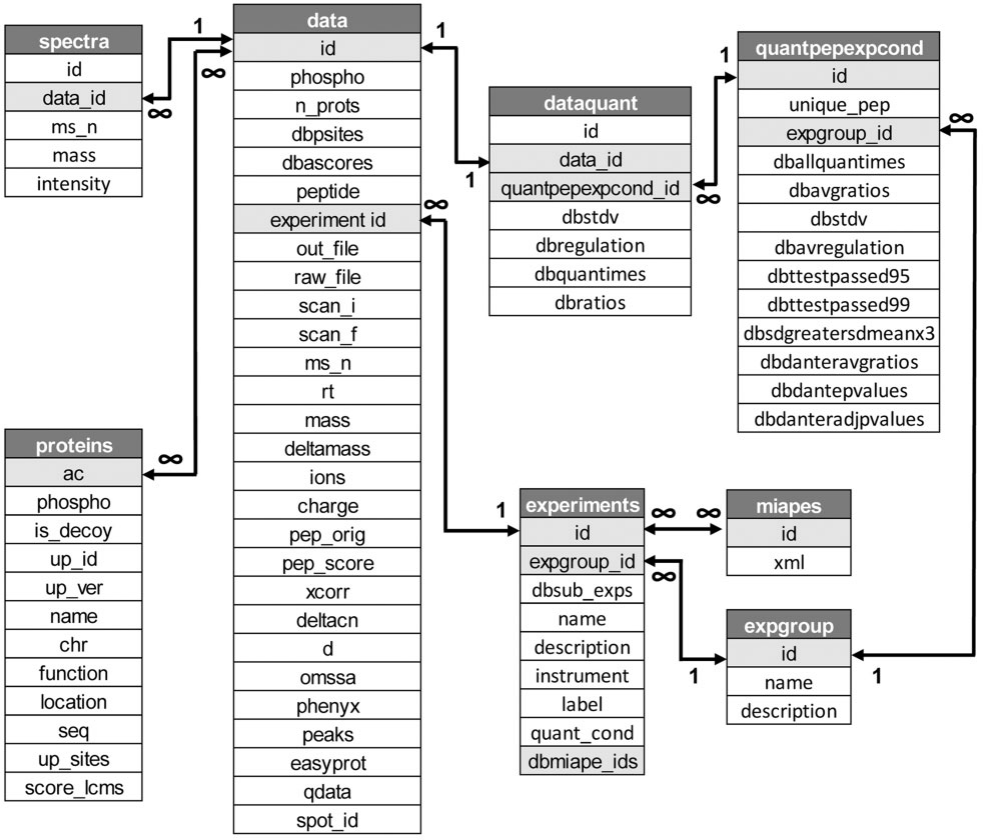
Schema of the database showing database main tables and fields (see Supplementary Figure S1 for details).

The structure and characteristics of the original database and web application was described in (6). Since then, the system has undergone significant changes with the implementation of new functions and features, especially those related with the visualization of quantitative data and the use of a sequence validation workflow based on the use of multiple search engines. As a consequence, the database schema has grown from six tables holding 36 parameters to 12 core and 5 auxiliary tables including a total of 117 fields (Figure 1 and Supplementary Figure 1). LymPHOS data has also been under constant review, with various content updates, bug fixes and additions of new experiments. Current data is derived from the analysis of primary T cells obtained from ca. 200 healthy donors along 31 different qualitative and quantitative experiments.

### Database contents

**Figure 2:**
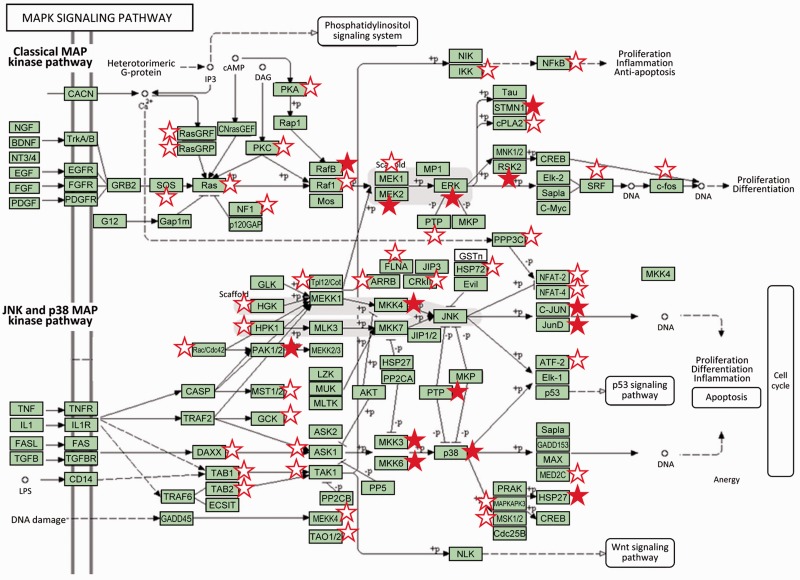
Classical and JNK/p38 MAP kinase pathways (modified from KEGG pathways). Proteins for which one or more sites were detected phosphorylated in resting cells or cells after TCR activation are marked with a star. Filled stars indicate the observation of a significant phosphorylation change after lymphocyte activation (P adj value < 0.05, 0.65  <  fold change  >  1.5). MAP kinases play a major role in the processing of the signals originated by different external stimuli which ultimately trigger cell responses such as proliferation, cell division and differentiation and apoptosis (17, 18). Components of the MAP kinase pathway are central on the signal transmission pathways triggered by activation of the lymphocyte T receptor including p38, which is activated by ZAP-70, or the components of the classical Ras/Raf/MEK/Erk MAP kinase cascade which activation involves the proteins SOS and RasGRP (upper part of the graphic). Activation by SOS is directed by its translocation through the GRB2-LAT complexes, while RasGRP is phosphorylated by PKC which in turn is activated by the DAG second messenger liberated by the activity of PLC-γ (18).

The new release of LymPHOS (http://lymphos.org, Table 1) contains a total of 8273 unique phosphopeptides corresponding to 2596 proteins (4937 when including isoforms). The database describes 15 566 phosphorylation sites, 10 608 characterized with high confidence (ratio of phosphorylation at Ser/Thr/Tyr: 88/11/1). These figures represent about a 45-fold increase over the original dataset in our 2009 publication [342 phosphorylation sites, (6)] and has allowed us to map lymphocyte key signaling pathways more extensively (Figure 2).

**Table:** Table 1. General information of LymPHOS database

Qualitative data	
Spectra	60 323
Unique phosphopeptides	8273
All p-sites	15 566
(pSer | pThr | pTyr)	(12 776 | 2494 | 296)
High-confidence p-sites	10 608
(pSer | pThr | pTyr)	(9288 | 1194 | 126)
Phosphoproteins^a^	4937/2596
Quantitative data	
Spectra	7990
Unique phosphopeptides	2315
All p-sites	4763
(pSer | pThr | pTyr)	(4169 | 545 | 49)
High-confidence p-sites	3959
(pSer | pThr | pTyr)	(3556 | 366 | 37)
Quantified phosphoproteins^a^	2272/1195

^a^All the possible proteins identified from the sequence peptides/Minimal group of proteins defining the sequenced peptides.

The most important improvements are related to data quality and the addition of quantitative data. We implemented a new workflow for peptide identification based on the use of several search engines in parallel to increase the number and confidence of identified sequences in our datasets (11). As the different search engines use different algorithms and scoring functions, identification of the same sequence by more than one search engine greatly increases the level of confidence in the match (19). Using this approach, individual search results can be generated and exported with minimal filtering. Assignations are then considered correct when identified by at least two search engines, a selection that is carried out by the Integrator application (12).

In addition, this version includes quantitative data for more than 2300 phosphopeptides containing 3959 high confidence p-sites which were quantified after activation at 15, 120 and 240 min (Table 2). Quantitative data has been obtained using the PQuantifier application. This application processes the Integrator output files (JSON format) along with information about the experimental conditions (cell treatment, labeling, activation times) to produce the averaged change ratios between controls and activated samples for each peptide. For each identified spectrum, PQuantifier assigns missing values, averages the intensities of duplicate reporter ions and calculates the ratios for the different time points relative to the control. For each experiment, PQuantifier normalizes these ratios relative to the distribution of non-phosphorylated peptides and calculates the peptide average ratios along the activation experiments.

**Table:** Table 2. Quantitative data included in the LymPHOS database. Data for the full database (All) and for the set of high confidence sequences (HC).

		Upregulated	Downregulated	No change
15 min	All	S: 1586 | T: 179 | Y: 12	S: 461 | T: 70 | Y: 6	S: 1734 | T: 241 | Y: 18
	HC	S: 1380 | T: 120 | Y: 9	S: 397 | T: 51 | Y: 6	S: 1486 | T: 155 | Y: 14
120 min	All	S: 1596 | T: 194 | Y: 12	S: 560 | T: 63 | Y: 5	S: 1625 | T: 233 | Y: 19
	HC	S: 1402 | T: 134 | Y: 9	S: 485 | T: 52 | Y: 5	S: 1376 | T: 140 | Y: 15
240 min	All	S: 617 | T: 45 | Y: 1	S: 56 | T: 2 | Y: 5	S: 421 | T: 50 | Y: 15
	HC	S: 545 | T: 34 | Y: 1	S: 55 | T: 2 | Y: 5	S: 366 | T: 45 | Y: 10

**Figure 3:**
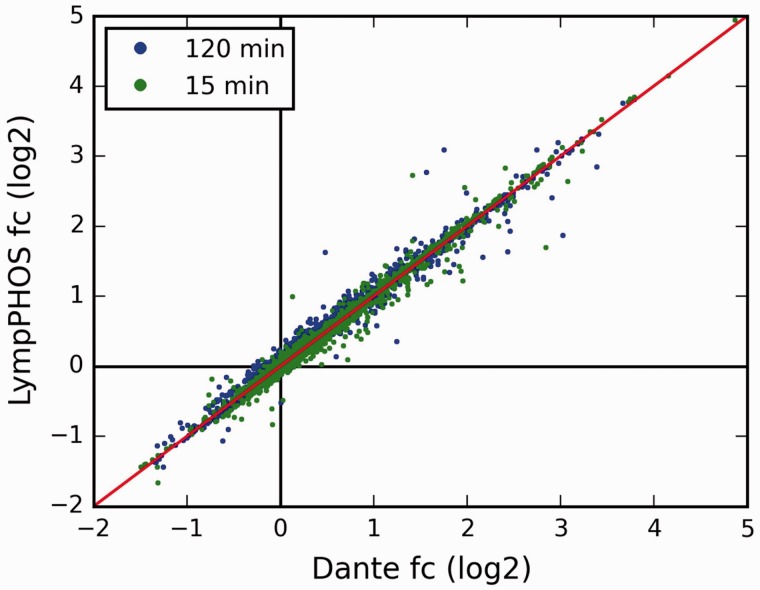
Comparison of the fold-change (fc) values obtained using the LymPHOS tools with those using the Dante package (18). For Dante analysis, each reporter ion had its values normalized against the median of non-phosphorylated peptide intensities. n  =  1562 for each time point, Coefficients of correlation r  =  0.98 (15 min) and r  =  1 (120 min).

**Figure 4:**
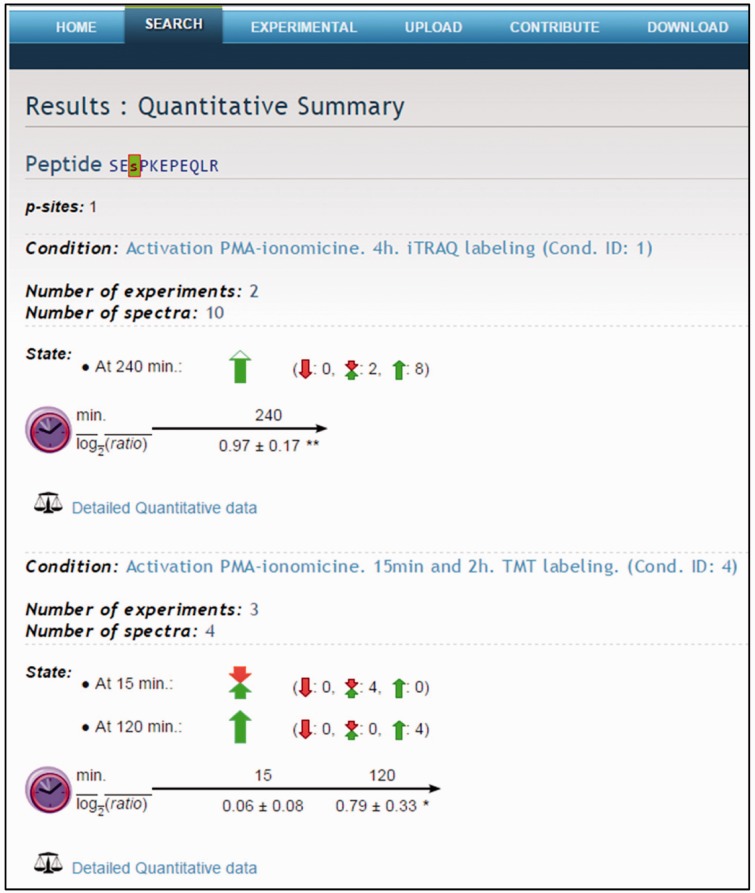
Quantitative summary. Example of the peptide SEsPKEPEQLR. Quantitative data for different p-sites is summarized using a visual presentation based on colored arrows.

**Figure 5:**
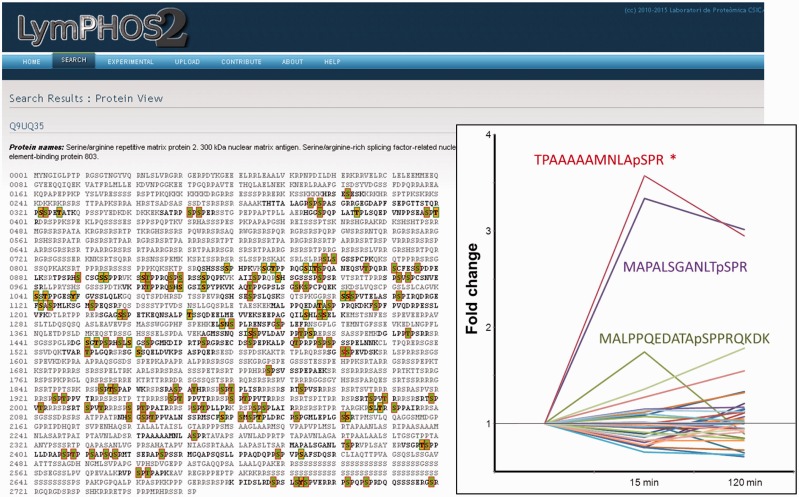
Protein view. Example of the protein Serine/Arginine repetitive matrix protein 2. More than 100 p-sites were identified for this protein of which only 1 showed statistically significant changes (insert). All phosphopeptides detected and quantified for the protein are shown in its sequence with green (high confidence) or red (low confidence) background.

Quantitative data produced with PQuantifier showed a good correlation with the corresponding data obtained using DanteR (20), a well-known software package with a different workflow for data processing (Figure 3). Major differences were observed in the quantitation from a few data points of bad quality (peptide quantified from a single spectrum and with many missing values). For example, when the two more biased outliers were removed from the set on 1543 points corresponding to the activation at 15 min, the coefficient of correlation increased from 0.9842 to 0.9998. The output of PQuantifier (JSON format), who uses itself a SQLite database for intermediary storage of the processed data, is then loaded into the MySQL database of the web application LymPHOS. The web application stores this quantitative information, creates the required links between quantitative data and the already stored mass spectrometry data, and performs dynamic calculations for the visual presentation of the observed changes. The quantitative data for different p-sites is summarized using a convenient, straightforward visualization that facilitates manual browsing and overviewing of the available results (Figure 4). All of the identified and quantified p-sites of a given protein are collected in the protein view (Figure 5).

**Figure 6:**
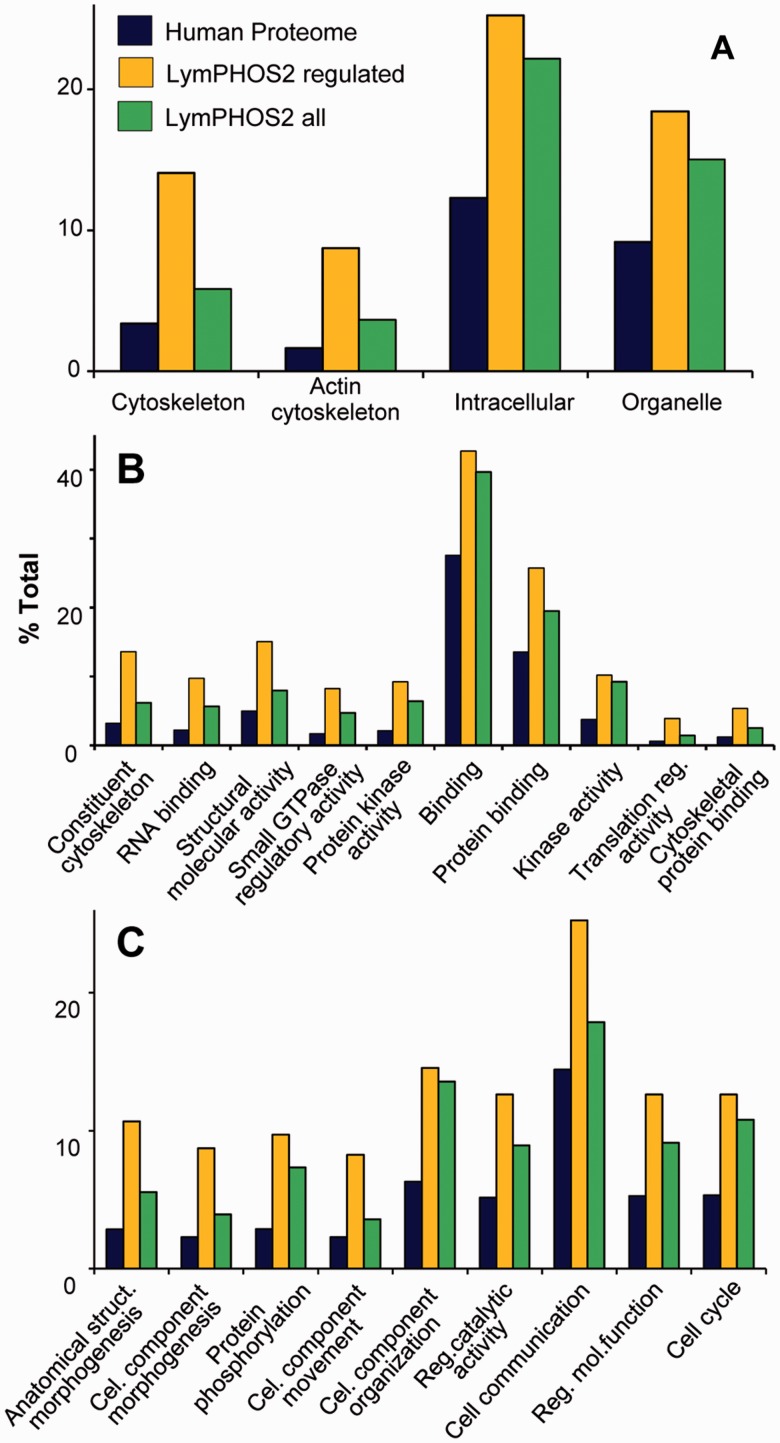
Gene Ontology (GO) enrichment analysis. Panther Database statistical overrepresentation test for the full LymPHOS phosphoprotein database (green) and the phosphoprotein set containing regulated p-sites (orange) versus the reference human proteome (blue). Classification uses GO-slim terms for cell compartment (A), biological process (B) and molecular function (C). Terms represented are those with enrichment > 1.5 and P  <  0.01 for the subset of regulated proteins. Terms are sorted (left to right) by increasing P value (http://pantherdb.org/).

Lymphocyte activation triggers intracellular signaling cascades, mostly regulated by protein phosphorylation/dephosphorylation events, which are ultimately responsible of cellular processes such as cell migration, endocytosis, cytokine liberation, proliferation, and apoptosis. An analysis of overrepresented GO terms in our database relative to the human proteome (Figure 6) showed the collection of regulated proteins (yellow bar) grouped into molecular functions and biological processes related to these cell activities. Proteins involved in signal transduction and cytoskeletal rearrangement were among the groups with a higher overrepresentation. Terms referring to signal transduction and phosphorylation processes include *kinase* and *protein kinase* activities and *protein phosphorylation* and *cell communication* processes. Morphological changes in the cell were represented by several GO terms related to cytoskeleton components, cytoskeletal binding proteins and morphogenesis functions. Thus, many non-motor and motor proteins associated to actin or the actin cytoskeleton (dynein, plastin, lamin, myosin, paxilin, vimentin, plextrin etc) were found regulated after cell activation. GO terms represented in Figure 6 were filtered taking into account its overrepresentation in the regulated subset of proteins. For all these terms, the proportion of proteins contributing from the full collection (green bar) was found smaller to that of the regulated proteins. This difference was mainly due to the subset of chromosome-associated phosphoproteins annotated in the database (grouped under the GO terms *protein–DNA complex*, *chromosome* and *chromosome-binding protein*) as well as to extracellular matrix proteins which are not regulated during activation (not shown).

### LymPHOS contribution to the human proteome project

The human proteome project is an international endeavor directed to the characterization of all the products encoded in the human genome. This includes the identification and quantification of proteins in healthy and pathological samples. HPP also aims to map specific PTMs of proteins. HPP efforts are distributed among the different partners on a chromosome per partner basis. In this context, LymPHOS data is being integrated on the HPP project through the Spanish Consortia which is in charge of the mining of chromosome 16 (21, 22). LymPHOS data contributes both to the description of new modification sites and of the proteome of a primary cell. Despite the difficulties inherent to the analysis of these cells in terms of availability and amount of protein obtainable, data obtained from primary cells is especially valuable as they would better represent the lymphocyte physiological states than modified cell lines.

The distribution of LymPHOS phosphoproteins based on their chromosome localization is similar to that found for the NextProt collection (23). The coverage of the protein-coding genome is of 10% and ranges from 7% to 14% depending on the chromosome. An exception is chromosome Y for which a coverage of only 4% was obtained (see Supplementary Figure S2). This could reflect the contribution of both male and female donors as well as the tissue specificity for the expression of many genes in this chromosome. Chromosome 16 (chr16) is represented with 90 phosphoproteins (52 of them with unique peptides) and 466 phosphopeptides.

## Conclusions

LymPHOS is an open access database for storage, sharing and visualization of data related with the human T-lymphocyte phosphoproteome. LymPHOS aims to provide a complete set of experimental data including chromatographic and spectrometric information. All MS^2^ and MS^3^ spectra justifying a p-site assignation are provided together with the corresponding Sequest, OMSSA, Phenyx, Peaks or EasyProt identification scores and p-site assignation scores (Q-Ascore). The web-based user interface allows searching phosphorylation sites on specific proteins and/or peptides as well as browsing the entire database, in all cases having experimental data to support each phosphorylation site assignment.

## Supplementary Data

Supplementary data are available at *Database* Online.

## Funding

This work was supported by project BIO2013-46492R from the Spanish Ministry of Economy and Competitiveness. T.D.N. was supported by a cooperation agreement between the Spanish National Research Council and the Vietnam Academy of Science and Technology (grant 2012VN0003). The CSIC/UAB Proteomics Facility belongs to ProteoRed and is partially funded by grants PT13/001 and PT13/008 from PRB2-ISCIII. Funding for open access charge: BIO2013-46492R.

*Conflict of interest*. None declared.

## Supplementary Material

Supplementary Data
