# Supplementary material for: LymPHOS 2.0: an update of a phosphosite database of primary human T cells
Source: Database (Oxford). 2015 Dec 23;2015:bav115. doi: 10.1093/database/bav115 (PMC4691341; doi:10.1093/database/bav115)
Supplement: Supplementary Data [file supp_2015_bav115_index.html]

LymPHOS 2.0: an update of a phosphosite database of primary human T cells — Supplementary Data 

# LymPHOS 2.0: an update of a phosphosite database of primary human T cells

## Supplementary Data

files

- Supplementary Data - pdf file
- Supplementary Data - pdf file
