# Supplementary material for: LymPHOS 2.0: an update of a phosphosite database of primary human T cells
Source: Database (Oxford). 2015 Dec 23;2015:bav115. doi: 10.1093/database/bav115 (PMC4691341; doi:10.1093/database/bav115)
Supplement: Supplementary Data [file supp_bav115_SI_Fig1_Rev1.pdf]

Supplementary Information Figure 1 (SI_Figure1).

Complete schema of LymPHOS2 database showing all tables and fields. Information in the database is arranged in five main groups of tables/classes:

Red and **Orange**: Main experimental data obtained by mass spectrometry and resulting from multiple proteomics search engines and data integration by the Integrator program.

Green: Protein data associated with the experimental data.

Yellow: Quantitative analysis data related to the experimental data, obtained after the statistical treatment and processing of the quantitative experimental data by the PQuantifier program.

Blue: Description of experiments, experimental groups and experimental procedures.

Gray: Accessory tables/classes related to the database management.

Updates from LymPHOS (Ovelleiro, D. et al. (2009) Proteomics, 9, 3741-3751):

All accessory tables are of new creation to facilitate the internal control (***lymphos_events***, ***lymphos_version***) and management (***lymphos_users***) of the database.

The database core has new tables with quantitative information:
- ***lymphos_dataquant***

Quantitative data for each spectrum in each experiment.
- ***lymphos_quantpepexpcond***

Statistics parameters of the quantitative measurements, for each peptide in each condition. Includes *t*-test outcome on the evaluation of a change, as well as the *p*-value and the adjusted *p*-value provided by DanteR analysis.

Former ***phosphosites*** table is now included in table ***lymphos_data***.

Former ***conditions*** table is now extended in three new tables:
- ***lymphos_experiments***

Basic experimental data, with a record for each experiment.
- ***lymphos_expgroup***

The experimental group table, or conditions table, to group closely related experiments.
- ***lymphos_miapes***

For the experimental information according to HUPO-PSI MIAPE guidelines.

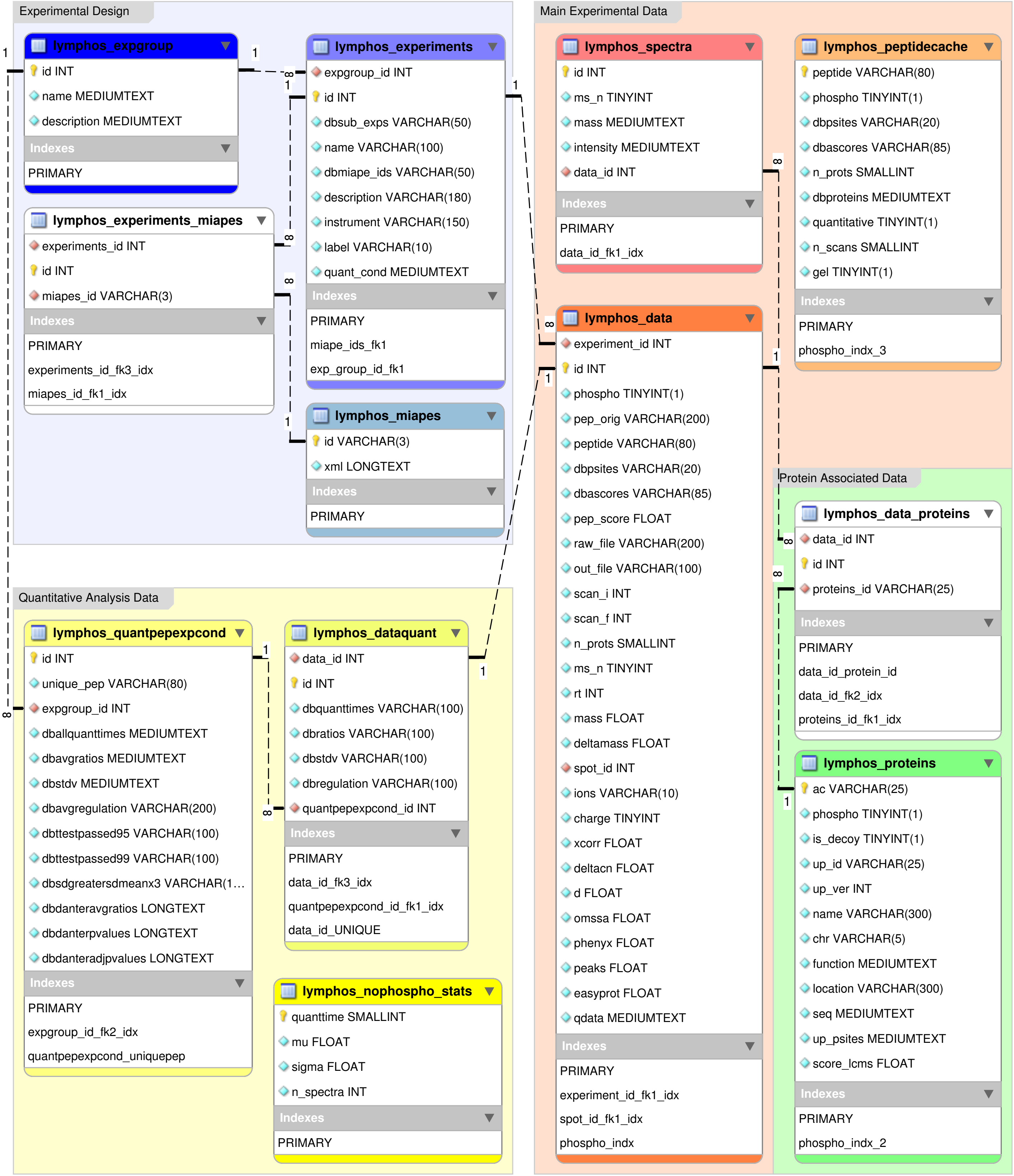

Experimental Design
lymphos_expgroup
id INT
name MEDIUMTEXT
description MEDIUMTEXT
Indexes
PRIMARY
lymphos_experiments
expgroup_id INT
id INT
dbsub_exps VARCHAR(50)
name VARCHAR(100)
dbmiape_ids VARCHAR(50)
description VARCHAR(180)
instrument VARCHAR(150)
label VARCHAR(10)
quant_cond MEDIUMTEXT
Indexes
PRIMARY
miape_ids_fk1
exp_group_id_fk1
lymphos_experiments_miapes
experiments_id INT
id INT
miapes_id VARCHAR(3)
Indexes
PRIMARY
experiments_id_fk3_idx
miapes_id_fk1_idx
lymphos_miapes
id VARCHAR(3)
xml LONGTEXT
Indexes
PRIMARY
Main Experimental Data
lymphos_spectra
id INT
ms_n TINYINT
mass MEDIUMTEXT
intensity MEDIUMTEXT
data_id INT
Indexes
PRIMARY
data_id_fk1_idx
lymphos_peptidecache
peptide VARCHAR(80)
phospho TINYINT(1)
dbpsites VARCHAR(20)
dbascores VARCHAR(85)
n_prots SMALLINT
dbproteins MEDIUMTEXT
quantitative TINYINT(1)
n_scans SMALLINT
gel TINYINT(1)
Indexes
PRIMARY
phospho_indx_3
lymphos_data
experiment_id INT
id INT
phospho TINYINT(1)
pep_orig VARCHAR(200)
peptide VARCHAR(80)
dbpsites VARCHAR(20)
dbascores VARCHAR(85)
pep_score FLOAT
raw_file VARCHAR(200)
out_file VARCHAR(100)
scan_i INT
scan_f INT
n_prots SMALLINT
ms_n TINYINT
rt INT
mass FLOAT
deltamass FLOAT
spot_id INT
ions VARCHAR(10)
charge TINYINT
xcorr FLOAT
deltacn FLOAT
d FLOAT
omssa FLOAT
phenyx FLOAT
peaks FLOAT
easyprot FLOAT
qdata MEDIUMTEXT
Indexes
PRIMARY
experiment_id_fk1_idx
spot_id_fk1_idx
phospho_indx
Protein Associated Data
lymphos_data_proteins
data_id INT
id INT
proteins_id VARCHAR(25)
Indexes
PRIMARY
data_id_protein_id
data_id_fk2_idx
proteins_id_fk1_idx
lymphos_proteins
ac VARCHAR(25)
phospho TINYINT(1)
is_decoy TINYINT(1)
up_id VARCHAR(25)
up_ver INT
name VARCHAR(300)
chr VARCHAR(5)
function MEDIUMTEXT
location VARCHAR(300)
seq MEDIUMTEXT
up_psites MEDIUMTEXT
score_lcms FLOAT
Indexes
PRIMARY
phospho_indx_2
Quantitative Analysis Data
lymphos_quantpepexpcond
id INT
unique_pep VARCHAR(80)
expgroup_id INT
dballquanttimes MEDIUMTEXT
dbavgratios MEDIUMTEXT
dbstdv MEDIUMTEXT
dbavgregulation VARCHAR(200)
dbttestpassed95 VARCHAR(100)
dbttestpassed99 VARCHAR(100)
dbsdgreatersdmeanx3 VARCHAR(1…
dbdanteravgratios LONGTEXT
dbdanterpvalues LONGTEXT
dbdanteradjpvalues LONGTEXT
Indexes
PRIMARY
expgroup_id_fk2_idx
quantpepexpcond_uniquepep
lymphos_dataquant
data_id INT
id INT
dbquanttimes VARCHAR(100)
dbratios VARCHAR(100)
dbstdv VARCHAR(100)
dbregulation VARCHAR(100)
quantpepexpcond_id INT
Indexes
PRIMARY
data_id_fk3_idx
quantpepexpcond_id_fk1_idx
data_id_UNIQUE
lymphos_nophospho_stats
quanttime SMALLINT
mu FLOAT
sigma FLOAT
n_spectra INT
Indexes
PRIMARY

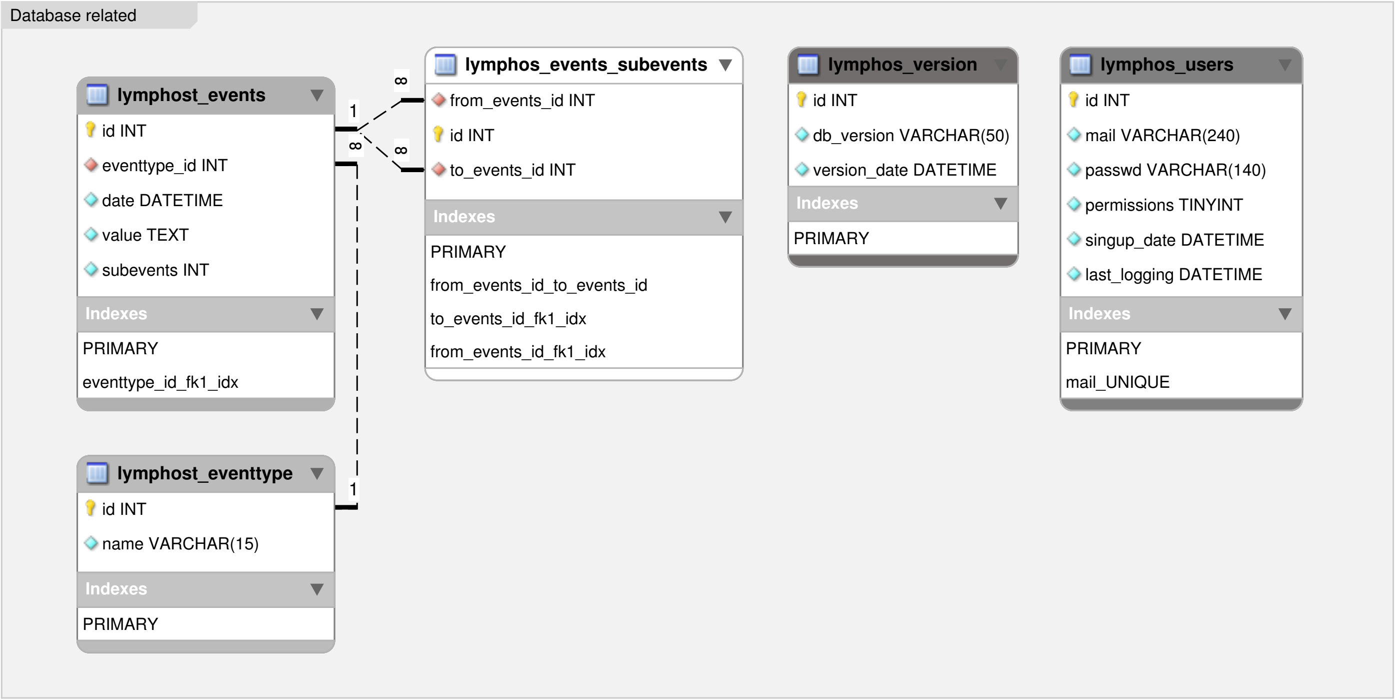

Database related
lymphost_events
id INT
eventtype_id INT
date DATETIME
value TEXT
subevents INT
Indexes
PRIMARY
eventtype_id_fk1_idx
lymphos_events_subevents
from_events_id INT
id INT
to_events_id INT
Indexes
PRIMARY
from_events_id_to_events_id
to_events_id_fk1_idx
from_events_id_fk1_idx
lymphos_version
id INT
db_version VARCHAR(50)
version_date DATETIME
Indexes
PRIMARY
lymphos_users
id INT
mail VARCHAR(240)
passwd VARCHAR(140)
permissions TINYINT
singup_date DATETIME
last_logging DATETIME
Indexes
PRIMARY
mail_UNIQUE
lymphost_eventtype
id INT
name VARCHAR(15)
Indexes
PRIMARY
1
∞
1
