# Supplementary material for: LymPHOS 2.0: an update of a phosphosite database of primary human T cells
Source: Database (Oxford). 2015 Dec 23;2015:bav115. doi: 10.1093/database/bav115 (PMC4691341; doi:10.1093/database/bav115)
Supplement: Supplementary Data [file supp_bav115_SI_Fig_2_rev1.pdf]

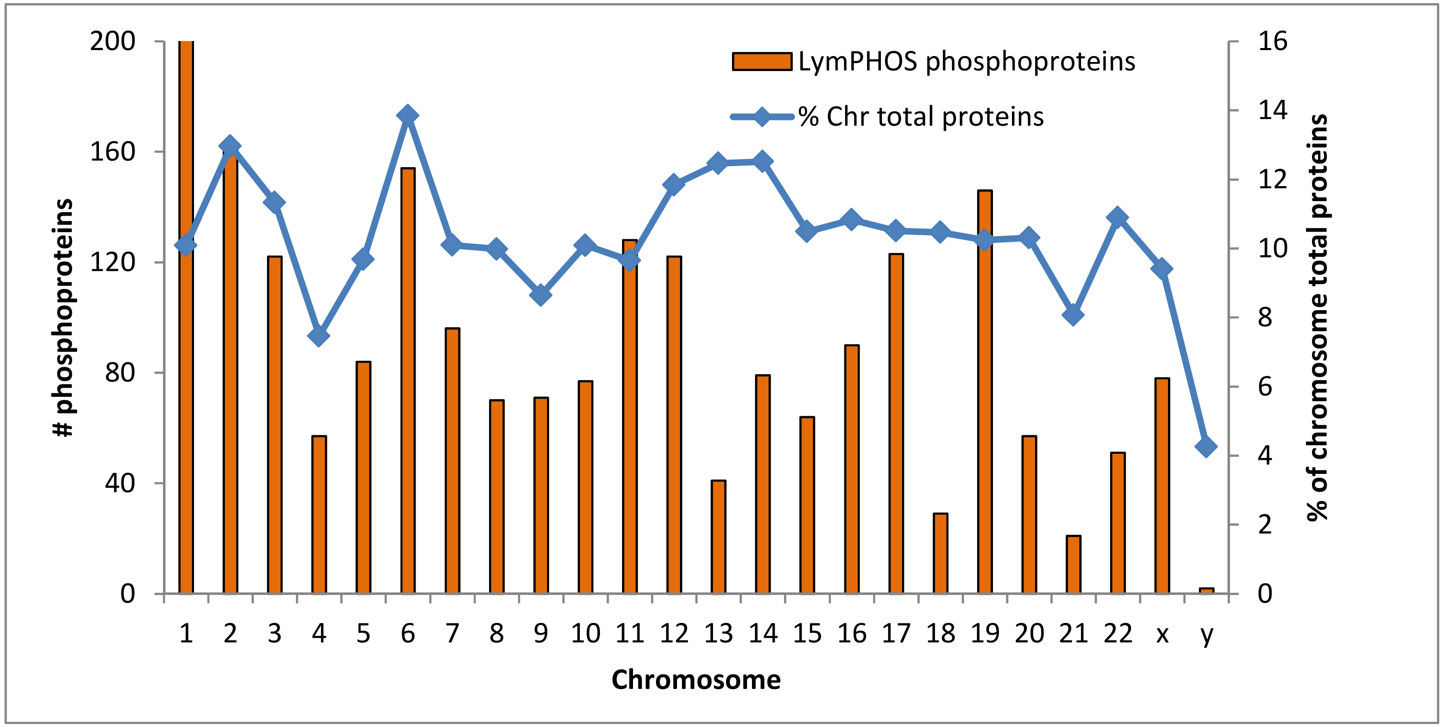

SI_Figure_2.- Classification of LymPHOS phosphoproteins based on chromosome localization. Blue dots indicate the percentage of LymPHOS phosphoproteins relative to the total number of proteins encoded in each chromosome.
